# Evaluation of the vector competence of a native UK mosquito *Ochlerotatus detritus* (*Aedes detritus*) for dengue, chikungunya and West Nile viruses

**DOI:** 10.1186/s13071-016-1739-3

**Published:** 2016-08-15

**Authors:** Marcus S. C. Blagrove, Ken Sherlock, Gail E. Chapman, Daniel E. Impoinvil, Philip J. McCall, Jolyon M. Medlock, Gareth Lycett, Tom Solomon, Matthew Baylis

**Affiliations:** 1 Department of Epidemiology and Population Health, Institute of Infection and Global Health, University of Liverpool, Liverpool, UK; 2 National Institute of Health Research Health Protection Research Unit in Emerging and Zoonotic Infections, University of Liverpool, Liverpool, UK; 3 Centers for Disease Control and Prevention, Atlanta, USA; 4 Vector Biology Department, Liverpool School of Tropical Medicine, Liverpool, UK; 5 Medical Entomology group, Emergency Response Department, Public Health England, Salisbury, UK

**Keywords:** DENV, WNV, CHIKV, Arbovirus, *Aedes*, *Ochlerotatus*, Mosquito, Vector competence

## Abstract

**Background:**

To date there has been no evidence of mosquito-borne virus transmission of public health concern in the UK, despite the occurrence of more than 30 species of mosquito, including putative vectors of arboviruses. The saltmarsh mosquito *Ochlerotatus detritus* [syn. *Aedes* (*Ochlerotatus*) *detritus*] is locally common in parts of the UK where it can be a voracious feeder on people.

**Methods:**

Here, we assess the competence of *O. detritus* for three major arboviruses: dengue virus (DENV), chikungunya virus (CHIKV) and West Nile virus (WNV) using adult mosquitoes reared from wild, field-obtained immatures.

**Results:**

We demonstrate laboratory competence for WNV at 21 °C, with viral RNA detected in the mosquito’s saliva 17 days after oral inoculation. By contrast, there was no evidence of laboratory competence of *O. detritus* for either DENV or CHIKV.

**Conclusions:**

To our knowledge, this is the first study to demonstrate competence of a UK mosquito for WNV and confirms that *O. detritus* may present a potential risk for arbovirus transmission in the UK and that further investigation of its vector role in the wild is required.

## Background

Although there have been 34 species of mosquito reported in the British Isles [1], including 12 known competent vectors of arboviruses elsewhere [2], no confirmed incidences of mosquito-borne virus transmission to humans has been recorded in the British Isles [3].

*Ochlerotatus detritus* [syn. *Aedes* (*Ochlerotatus*) *detritus*] is abundant throughout coastal regions of the British Isles, with immature mosquitoes commonly found in coastal brackish waters, particularly those prone to flooding at both the spring high tide zone and in regularly flooded saline lagoons [4, 5]. *Ochlerotatus detritus* is a multivoltine species, producing large populations following each spring flood of areas where eggs have previously been deposited awaiting saline submergence. Eggs of *O. detritus* can survive for over a year [6], with peak adult activity occurring between March and November when, in coastal areas, they are often the greatest biting nuisance of any British mosquito [7]. Although it is primarily a coastal species, there is evidence of populations inland in freshwater habitats [8]. *Ochlerotatus detritus* has a highly catholic feeding behaviour, commonly feeding on humans, birds and livestock [7], thus also making it a potential bridge vector of many zoonotic arboviruses. It has been implicated as the most common nuisance biting species of humans in England [9] and the most common mosquito of newly created coastal habitat [5].

With increasing global travel of both humans and livestock, as well as changing global climatic conditions, the geographic range of many mosquito-borne arboviruses has been increasing in recent decades. The most prominent examples of this phenomenon are West Nile virus (WNV), dengue virus (DENV) and chikungunya virus (CHIKV).

WNV has expanded its range from a small area of sub-Saharan Africa to the six major continents in the last 25 years [10]. Outbreaks of WNV in Europe occur annually and given that the virus can be moved around the continent in migratory birds, there would appear to be a route of entry for the virus in the United Kingdom (UK). Furthermore, WNV occurs in regions in similar climatic conditions to the UK such as Canada [11]. Moreover, antibodies have been detected in migratory and domestic birds in the UK [12, 13] indicating that the UK may be at risk of the establishment of WNV.

The incidence of new dengue cases globally is estimated to have increased 30-fold in the last 50 years [14, 15]. This can in part be attributed to an increase in the geographical range of the virus and an increase in the human population within, and travelling to, high risk areas [16]. The geographical range of CHIKV has also increased over a similar time period, with more recent expansions believed to be the result of a range of novel mutations increasing the replication rate in *Ae. albopictus* [17]. Furthermore, there has also been a significant expansion of CHIKV to the Americas with a large outbreak (> 1 million cases) in the Caribbean region. The occurrence of these viruses circulating in geographical regions where there are increased numbers of UK travellers poses a potential risk for the virus to spread to the UK through infected travellers [3]. The range expansion of both of these viruses has included an extension into regions with cooler climates, including sporadic autochthonous transmission as far north as France, highlighting the potential future risk to the UK [18, 19].

Previously, our group has demonstrated that field-collected *O. detritus* are competent laboratory vectors of Japanese encephalitis virus (JEV), showing potential for transmission by the mosquito at 7 days post-infection at 23 °C [20]. Given this, as well as the high abundance and biting nuisance of *O. detritus*, it is important to determine whether *O. detritus* is a laboratory-competent vector of the aforementioned invasive arboviruses in order to determine the risk to the UK from this potential vector. In this study, we attempted to infect *O. detritus* experimentally with three of the most globally important and invasive arboviruses, WNV, DENV and CHIKV, in order to determine the vector competence of this species.

## Methods

*Ochlerotatus detritus* immatures (fourth-instar larvae and pupae) were collected from marshland by Little Neston, Cheshire, UK (GPS coordinates: 53°16′37.2″N, 3°04′06.4″W). Immatures were collected using a fine scrim net and non-mosquitoes were removed from the sample using a Pasteur pipette. Immatures were reared in ambient conditions in water collected from their larval habitat until adulthood; no additional food source was provided in order to ensure the mosquitoes remained as representative of the wild population as possible. Adults were allowed to emerge and mate in 30 × 30 × 30 cm BugDorms (BugDorm, Taichung, Taiwan). Control colony *Ae. aegypti* (New Orleans strain) (DENV and CHIKV) and *Cx. quinquefasciatus* (Recife strain) (WNV) were used for comparison. Colony mosquitoes were reared in an insectary at 25 °C 12:12 light:dark photoperiod and 70 % relative humidity (RH).

At seven days post-emergence, female adults were removed and transferred into 1 l cylindrical polypropylene DISPO-SAFE containers, with a fine mesh covering the container opening and stored for 24 h with no access to sugar. Blood meals (heparinised human blood, NHS transfusion service, Speke) containing virus (or blood only control) were provided for 3 h with an odorised feeding membrane. Unfed adults were removed from the cage, and the fed mosquitoes were incubated at 21 °C and 70 % RH for 17 days. Mortality was recorded 48 h and 17 days after feeding. 21 °C was used as this approximates a very hot summer in the south east of England. 17 days (as opposed to the standard 14 days) was used to counter the likely lengthy increase in extrinsic incubation period as a result of the relatively low experimental temperatures.

The virus strains were as follows: WNV NY-99, cultured at Public Health England, Porton Down, Surrey, in Vero cells; DENV Serotype 2, Bangkok Thailand; CHIKV NC/2011-568 (CHIKV_NC) cultured by the Brain Infections Group, University of Liverpool, in Vero cells. Final titres of virus in blood were as follows: WNV 2 × 10^6^ PFU/ml; CHIKV 1 × 10^7^ PFU/ml; DENV 1 × 10^7^ PFU/ml, titres were limited by the available stock concentration provided by the respective institutions. Virus strains were confirmed by sequencing prior to experimentation.

On day 17, mosquitoes were anaesthetised with FlyNap (Carolina Biological Supply Company, Burlington, North Carolina, USA), and their saliva was extracted by inserting their proboscis into a capillary tube containing mineral oil. RNA was extracted from the expectorate using TRIzol reagent (Thermo Fisher Scientific). cDNA was generated using Superscript Vilo (Thermo Fisher Scientific).

An additional experiment was performed for DENV using different conditions in an attempt to establish the cause of mortality in *O. detritus* fed with this virus. A 1/100 concentration condition was produced by serial dilution with blood, and a deactivated virus condition was produced by heating the virus (prior to adding to blood) to 70 °C for 10 min in a water bath.

Taqman (Thermo Fisher Scientific) quantitate reverse transcription polymerase chain reaction (qRT-PCR) was used to detect the presence of viral RNA in the samples. Primer and probe sets were as follows: WNV, sense 5′-CCA CCG GAA GTT GAG TAG ACG-3′, anti-sense 5′-TTT GGT CAC CCA GTC CTC CT-3′, probe Cy5-TGC TGC CTG CGG CTC AAC CC-BBQ, regimen 1 min at 95 °C followed by 40 cycles of 95 °C for 5 s and 60 °C for 8 s [21]. CHIKV, sense 5′-GCA TCA GCT AAG CTC CGG GTC-3′, anti-sense 5′-CAA TGT CTT CAG CCT GGA CAC C-3′, probe Cy5-ATG CAA ACG GCG ACC ATG CCG TCA-BBQ, regimen 95 °C for 2 min followed by 40 cycles of 95 °C for 15 s, 55 °C for 10 s, 60 °C for 10 s and 72 °C for 20 s [22]. DENV, sense 5′-GAC TAG YGG TTA GAG GAG ACC-3′, anti-sense 5′-GHR GAG ACA GCA GGA TCT CTG-3′, probe JOE-AAG GAC TAG MGG TTA GWG GAG ACC C-BBQ, regimen 95 °C for 2 min followed by 40 cycles of 95 °C for 15 s, 55 °C for 10 s, 60 °C for 10 s and 72 °C for 20 s [22]. Positive controls (neat virus) and negative control (neat blood) were performed alongside all qRT-PCR experiments.

## Results

No virus-positive expectorate was recorded for *O. detritus* with CHIKV, although 61 % of *Ae. aegypti* were found to be virus-positive (Table 1). No significant difference was found between the mortality rate of *O. detritus* and *Ae. aegypti* infected with CHIKV at 48 h (Fisher’s exact test, two-tailed, *P* = 0.529). However, the mortality rate of *O. detritus* was significantly higher at 17 days (Fisher’s exact test, two-tailed, *P* = 0.001). Blood only controls from the DENV experiment below were qRT-PCR tested for CHIKV as a negative control; all individuals tested negative.

**Table 1:** Mortality and competence of *Ochlerotatus detritus* for CHIKV

Species	No. of mosquitoes fed	Mosquito mortality at 48 h (%)	Mosquito mortality at 17 day (%)	No. of fed mosquitoes positive (%)	Percentage of mosquitoes positive^a^	Percentage of surviving mosquitoes positive^b^
*O. detritus*	143	13 (9.1)	41 (28.7)	0	0	0
*Ae. aegypti*	158	11 (7.0)	21 (13.3)	83	52.5	60.6

^a^Number of positive/Total number of mosquitoes at 17 days

^b^Number of positive/Number of mosquitoes alive at 17 days

In the first replicate of the DENV experiment, within 48 h, 98 % of all blood-fed female *O. detritus* had died (whilst almost no unfed-females died), compared to 3 % mortality in *Ae. aegypti* (Fisher’s exact test, two-tailed, *P* < 0.0001). The experiment was repeated using four conditions: full concentration DENV (replicate 2); 1/100 dilution of virus; a heat deactivated full concentration virus; and blood only (Table 2). 48 h mortality was again very high (> 90 %) with full concentration DENV (replicate 2), and again significantly higher than that of *Ae. aegypti* (Fisher’s exact test, two-tailed, *P* < 0.0001); no significant difference was found between the two full concentration replicates (Fisher’s exact test, two-tailed, *P* = 0.4863). The 48 h mortality rate was significantly reduced by the virus being diluted to 1/100 concentration (Fisher’s exact test, two-tailed, *P* < 0.0001), as well as by the virus being heat-deactivated (Fisher’s exact test, two-tailed, *P* < 0.0001). There was no significant difference between 48 h mortalities with deactivated or diluted virus (Fisher’s exact test, two-tailed, *P* = 0.580); however, both deactivated and diluted virus cause significantly higher mortality at 48 h compared to the no virus (blood only) control (Fisher’s exact test, two-tailed, *P* = 0.0006 and *P* < 0.0001, respectively).

**Table 2:** Mortality and competence of *Ochlerotatus detritus* for DENV

Species	Condition	No.quitoes fed	Mosquito mortality at 48 h (%)	Mosquito mortality at 17 day (%)	No. of fed mosquitoes positive	Percentage of mosquitoes positive^a^	Percentage of surviving mosquitoes positive^b^
*O. detritus*	Full rep 1	982	959 (97.7)	982 (100)	0	0	0
	Full rep 2	93	90 (96.8)	93 (100)	0	0	0
	1/100	102	21 (20.6)	34 (33.3)	0	0	0
	Deactivated	89	15 (16.9)	28 (31.5)	0	0	0
	Blood only	94	2 (2.1)	17 (18.1)	0	0	0
*Ae. aegypti*	Full	207	6 (2.9)	25 (12.1)	127	61.4	69.8

^a^Number of positive/Total number of mosquitoes at 17 days

^b^Number of positive/Number of mosquitoes alive at 17 days

Twenty-one % of the surviving *O. detritus* blood-fed females at 17 days were virus-positive for WNV, compared to 53 % *Cx. quinquefasciatus* (Table 3) (Fisher’s exact test, two-tailed, *P* < 0.0001). The mortality rate of *Cx. quinquefasciatus* was significantly higher than that of *O. detritus*, when infected with WNV, at both 48 h (Fisher’s exact test, two-tailed, *P* < 0.0001), and 17 days (Fisher’s exact test, two-tailed, *P* < 0.0001). Blood only controls from the DENV experiment below were qRT-PCR tested for WNV as a negative control; all individuals tested negative.

**Table 3:** Mortality and competence of *Ochlerotatus detritus* for WNV

Species	No. of mosquitoes fed	Mosquito mortality at 48 h (%)	Mosquito mortality at 17 day (%)	No. of fed mosquitoes positive (%)	Percentage of mosquitoes positive^a^	Percentage of surviving mosquitoes positive^b^
*O. detritus*	89	2 (2.2)	11 (12.4)	16	17.9	20.5
*Cx. quinquefasciatus*	143	26 (18.2)	53 (37.1)	48	33.6	53.3

^a^Number of positive/Total number of mosquitoes at 17 days

^b^Number of positive/Number of mosquitoes alive at 17 days

**Fig. 1:**
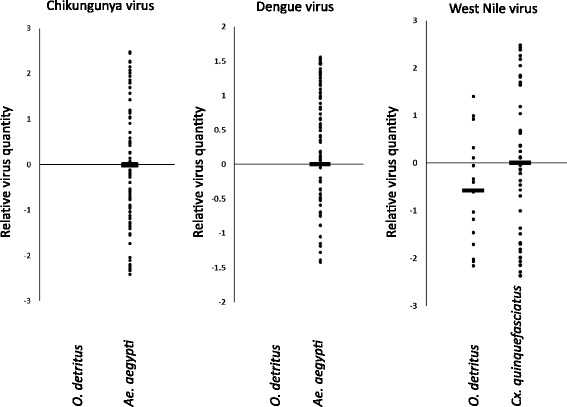
Relative quantity of viral RNA in expectorate determined by qRT-PCR. All virus-positive results are shown as a quantity relative to the mean titre of the control vector (*Ae. aegypti* for CHIKV and DENV; and *Cx. quinquefasciatus* for WNV). Horizontal bars represent the mean

The relative quantities of virus for each experiment are shown in Fig. 1 (multiple entries for *O. detritus* and DENV were not made as all equalled zero). There was no significant difference between the relative quantity of WNV virus recovered from the expectorate of *O. detritus* and *Cx. quinquefasciatus* (Wilcoxon rank sum test, two tailed, *P* = 0.1674).

## Discussion

In this study the abundant, but locally restricted, British mosquito *O. detritus* was assessed for its competence for DENV, CHIKV and WNV; *O. detritus* was shown to be competent for only WNV. To our knowledge, this is the first demonstration that a wild-caught British mosquito is laboratory-competent for WNV and the first demonstration that *O. detritus* is laboratory-competent for WNV.

Our results raise the question as to whether *O. detritus* may be an efficient vector for WNV in the wild with the potential to sustain local transmission of WNV in the event of introduction of the virus to the UK. Furthermore, there was no significant difference between the amount of virus recovered from the expectorate of *O. detritus* and the known vector *Cx. quinquefasciatus*. Given both the catholic feeding habits of *O. detritus* (feeding on birds, livestock and humans) and the evidence presented here of its laboratory competence for WNV transmission, we suggest that the role of *O. detritus* as a potential risk for WNV reservoir circulation in birds, transmission to humans and transmission to horses requires consideration. WNV is considered to have been introduced into North America by either migratory birds or exotic birds transported via aeroplane [23]. Such a method of introduction is possible in the UK [24], indeed, there has been evidence of WNV antibodies in migratory and domestic birds in the UK, suggesting that invasion is possible [12, 13]. Given this and the expansion of the range of WNV to areas with similar climates to the UK, it would appear that the UK may be at risk of WNV introduction and circulation. Important next steps in the analysis of risk from this vector/virus combination are an assessment of the effects of temperature and viral titre on competence in order to determine the likelihood of virus transmission and whether UK temperatures would be sufficient to sustain it.

In contrast to the competence of *O. detritus* for WNV, our data show that the mortality of *O. detritus* may be greatly increased by oral infection with DENV. We found a highly significant increase in the mortality of *O. detritus* compared to *Ae. aegypti* when using full concentration virus. However, we also showed that ‘deactivating’ the virus did not completely negate the increased mortality, and that there was no significant difference between low titre and deactivated virus. These findings could also be consistent with a contaminant causing mortality (however, no unusual mortality was observed with *Ae. aegypti*). This effect of DENV on *O. detritus* warrants further study; whilst it does not prove that DENV is causing the mortality, it is consistent with this theory and similar effects have been noted in some previous studies. Whilst most studies have shown no fitness costs of DENV to its host, e.g. [25], there are some previously described incidences of arboviruses causing fitness costs to their vectors, including reduced longevity of *Ae. aegypti* infected with DENV [26–28]. Reduced longevity has also been observed with other arboviruses such as Western equine encephalitis virus (WEEV) and Eastern equine encephalitis virus (EEEV) [29, 30]. These studies however, show a relatively minor effect on longevity compared to the data presented here. This is likely the result of previous studies focusing on the fitness effects of arboviruses on their natural vectors; given the geographical ranges of DENV and *O. detritus*, it is extremely unlikely that DENV has adapted to minimize any negative fitness effects on *O. detritus*. Our data are therefore consistent with the prevailing theory that arboviruses adapt to minimize their effect on the longevity of their natural vectors and may have significantly greater fitness effects on non-natural vectors [31].

Given both the extreme effect on longevity, together with the lack of any virus in the expectorate of *O. detritus* in the low-dose DENV condition, it seems highly unlikely that *O. detritus* will pose a significant future risk of DENV transmission in the UK.

Our results also show no evidence for vector competence of *O. detritus* for CHIKV. Unlike the DENV infection however, CHIKV caused no significant mortality. The colony *Ae. aegypti* control infection did produce infectious females at 17 days at 21 °C, ruling out inactive virus. Whilst it is not known whether higher temperatures or increased time to expectorate extraction would produce infectious *O. detritus*, the failure to detect any CHIKV despite the long incubation period provides no evidence for risk of CHIKV transmission from this population of *O. detritus*.

## Conclusions

In addition to our previous work showing competence of *O. detritus* for JEV [20], here, we have shown that there appears to be no evidence to suggest that there is a risk to the UK from *O. detritus* vectoring either DENV or CHIKV, but in contrast there is a potential risk in its role as a putative WNV vector. *Ochlerotatus detritus* is a nuisance mosquito species in the UK, and can be highly abundant in some coastal habitats. Given both the competence and feeding habits of *O. detritus*, this species may pose a credible threat for transmission of both JEV and WNV. To our knowledge, this is the first time wild UK mosquitoes have been demonstrated to be laboratory competent for WNV. However, further work is required to understand whether this laboratory competence translates into a risk of transmission in natural environments.

## Abbreviations

Not applicable.
